# A quantile regression forest based method to predict drug response and assess prediction reliability

**DOI:** 10.1371/journal.pone.0205155

**Published:** 2018-10-05

**Authors:** Yun Fang, Peirong Xu, Jialiang Yang, Yufang Qin

**Affiliations:** 1 Department of Mathematics, Shanghai Normal University, Shanghai, China; 2 School of Mathematics and Statistics, Hainan Normal University, Haikou, China; 3 Department of Genetics and Genomic Sciences, Icahn School of Medicine at Mount Sinai, New York, NY, United States of America; 4 College of Information Technology, Shanghai Ocean University, Shanghai, China; Roswell Park Cancer Institute, UNITED STATES

## Abstract

Drug response prediction is a critical step for personalized treatment of cancer patients and ultimately leads to precision medicine. A lot of machine-learning based methods have been proposed to predict drug response from different types of genomic data. However, currently available methods could only give a “point” prediction of drug response value but fail to provide the reliability and distribution of the prediction, which are of equal interest in clinical practice. In this paper, we proposed a method based on quantile regression forest and applied it to the CCLE dataset. Through the out-of-bag validation, our method achieved much higher prediction accuracy of drug response than other available tools. The assessment of prediction reliability by prediction intervals and its significance in personalized medicine were illustrated by several examples. Functional analysis of selected drug response associated genes showed that the proposed method achieves more biologically plausible results.

## Introduction

Identifying individual therapy for cancer patients to maximize drug efficacy is a fundamental and key step towards precision medicine. In theory, the efficacy of a drug depends on a variety of factors including molecular, clinical and environmental features of a patient sample. So, given a set of samples with drug response data, it is feasible to design machine-learning methods to predict drug response values from different types of genetic and clinical features.

One of the earliest attempts in this task could trace back to the NCI60 data set, which consists of gene expression profiles of 60 human cancer cell lines with different tissue origin and cytotoxicity profiles of >100,000 chemical compounds [1]. Ever since then, a number of methods have been proposed to predict drug response based on gene expression profiles. For example, Riddick *et al*. built an ensemble regression model using Random Forest [2]; Lee *et al*. developed a co-expression extrapolation algorithm by comparing the differences of gene expression between sensitive and resistant cell lines [3]. However, one important drawback of the NCI60 dataset is its small sample size, which potentially leads to many false positive associations between gene expression and drug response. With the advent of high-throughput techniques, scientists are able to monitor drug responses and genomic features of large number of samples in parallel with affordable cost. For example, two consortiums, the Cancer Cell Line Encyclopedia (CCLE) [4] and Cancer Genome Project (CGP) [5], collectively analyzed around 1,000 clinically-relevant human cell lines and their pharmacological profiles for 149 cancer drugs. These two studies also included the gene expression profiles and mutation status for each cell line, and applied a sparse linear regression model, *i*.*e*., the elastic net, to select expression and mutation signatures that are predictive of drug responses. Based on the same dataset, Geeleher *et al*. applied another sparse regression model, the Ridge regression, to predict drug response for breast cancer cell lines using baseline gene expression data [6]. Fang *et al*. applied the iterative sure independence screening (ISIS) integrated with lasso to predict the activity area of 24 anti-cancer drugs [7]. Wan and Pal utilized the top features in different genomic characterizations via an integrated random forest model [8].

All the aforementioned methods focused on point prediction via the conditional mean (expectation) of drug responses, with little discussion on their prediction intervals (PIs). For example, mean squared error (MSE) and the correlation between predicted and observed values can be used to quantify the accuracy of point prediction, however, they give little information on the possible fluctuation of the drug response for each sample. In contrast, prediction interval not only gives a range where the drug response locates in with high probability but also provides the prediction reliability by its length simultaneously. At the same confidence level, the shorter interval indicates more reliable prediction. So in precision medicine, besides the extensively studied point prediction, prediction interval for drug response is also helpful [9].

**Fig 1:**
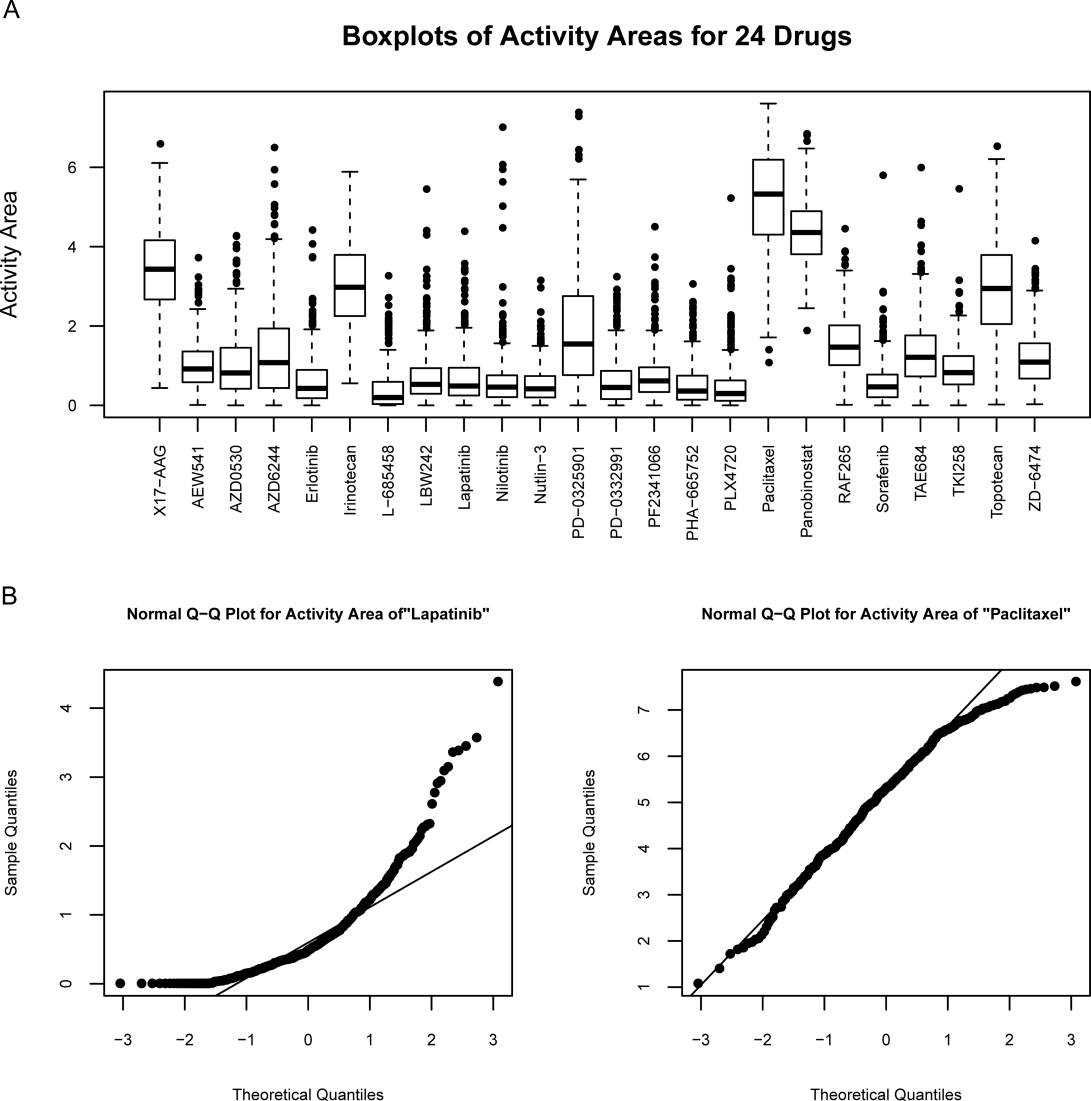
Boxplots and normal Q-Q plots of the activity areas in the CCLE dataset. Panel (A) shows the boxplots of activity areas for 24 drugs. Panel (B) shows the normal Q-Q plots of activity area for two example drugs Lapatinib and Paclitaxel.

Under the normality assumption on the drug response or random error, it is not difficult to estimate the variance of predicted values and further derive prediction intervals by traditional regressions. However, as suggested by the boxplots and Q-Q plots in Fig 1, activity areas (drug responses) for most drugs in the CCLE dataset do not follow normal distribution. To solve this problem, we can resort to quantile regression [10] which has been studied extensively in many fields including finance, sociology, immunology, etc. [11–15]. Quantile regression does not require to assume a specified distribution for drug response. Moreover, different from least squares which only fit one curve (the conditional mean of drug response given genomic features [4,7]), quantile regression can fit a bunch of curves (conditional quantiles) thus generate a more comprehensive characterization of drug response. Consequently, prediction intervals for drug response can be constructed by the predicted quantiles.

In this paper, we proposed a three-step quantile regression forest (QRF) approach for predicting the drug responses and applied it into the CCLE dataset. To capture potentially important features and reduce noise, we firstly implemented primary feature screening and then trained a random forest for variable selection. Consequently, we obtained point predictions based on the mean and median of predicted drug response by QRFs. At the same time, we constructed prediction intervals to assess the prediction reliability. To further compare drug responses when point predictions are the same, we also tested the homogeneity of variances based on the Levene test, and gave two examples to state the significance of reliability prediction. Finally, we annotated the selected drug response associated genes by David tools (https://david.ncifcrf.gov/summary.jsp) and showed more biologically meaningful results.

## Materials and methods

### Data resources

In this paper, we used the cancer genomic and drug response data from the Cancer Cell Line Encyclopedia (CCLE). The CCLE dataset (http://www.broadinstitute.org/ccle) consists of large-scale genomics data, including expression profile of 20089 genes, mutation status of 1667 genes, copy number variation of 16045 genes for 947 human cancer cell lines, and 8-point dose-response curves for 24 chemical drugs across 479 cell lines. Drug sensitivities to a given cell line are evaluated by IC50, EC50, and activity area (the area over dose-response curves). In this study, activity area is used as a drug sensitivity measurement due to its ability to capture the efficacy and potency of drug sensitivity simultaneously compared to IC50 and EC50.

### Method overview

**Fig 2:**
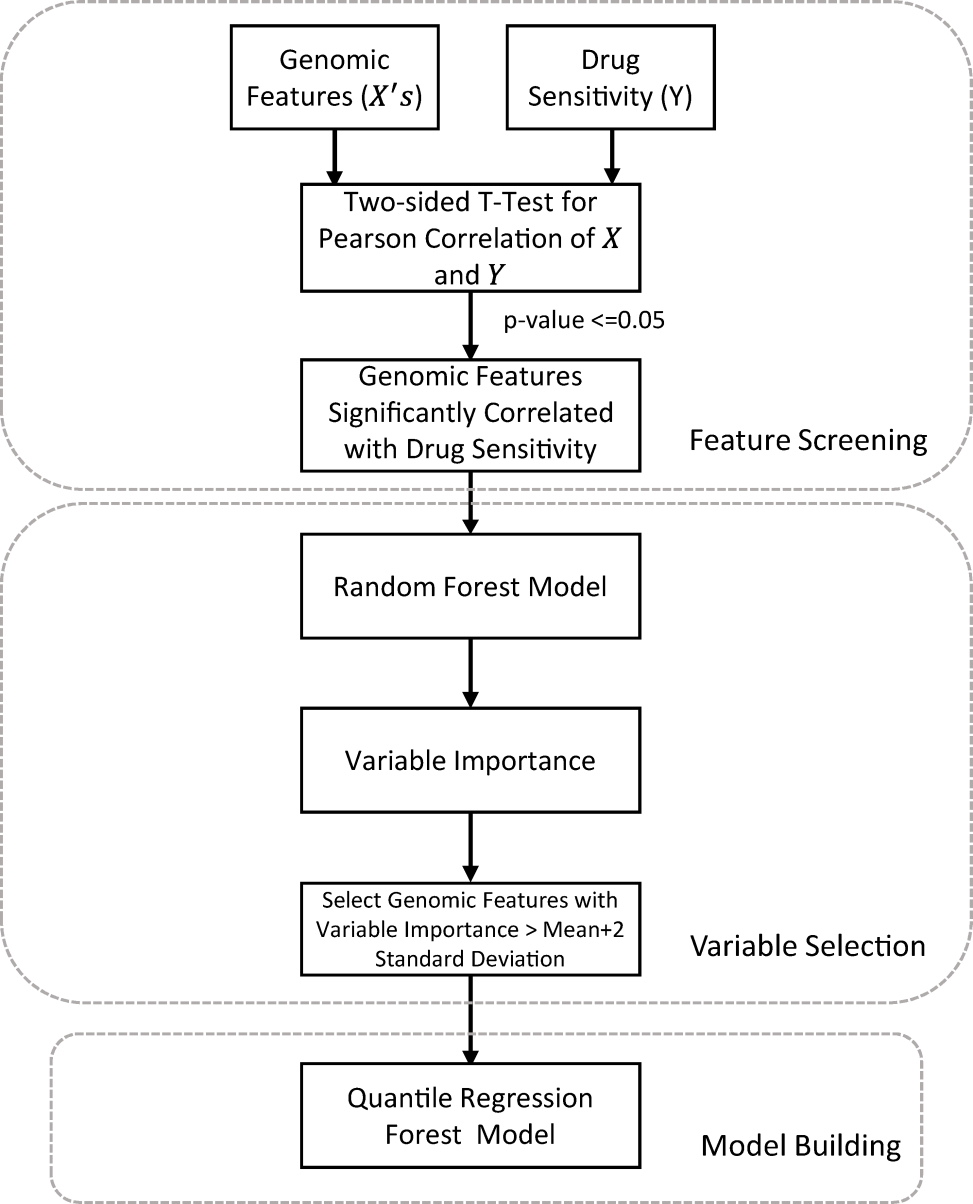
Workflow of the three-step quantile regression forest method. All features were screened by their Pearson correlations with drug response. Then a random forest was trained to rank selected features by their importance. The variables with the importance of twice standard deviation greater than the mean of importance were selected for the final quantile regression forest.

QRFs for 24 chemical compounds were trained based on three types of genomic features, including gene expressions, mutation status and copy number variation. Motivated by Riddick *et al*. [2], we designed a three-step quantile regression forest method as follows (Fig 2). In the first stage, some important genomic features were filtered through the correlation test. Then, a random forest was trained on the filtered features and variables are ranked and furtherly selected based on their importance. Finally, QRF is built using selected features.

### Feature screening by Pearson correlation coefficient

Due to the ultra-high dimension of genomic features in CCLE dataset, building QRFs directly from all available features is difficult and time-consuming. So a screening method was first applied to filter the potentially important features [16]. We calculated the Pearson correlation coefficients (PCCs) of genomic features with drug responses and ranked the importance of features by p-values of two-sided t-tests for PCCs. Features with p-value under 0.05 were then selected. For each drug in CCLE, the above feature screening process ranked the marginal importance of genomic features and selected around 2000 genes.

### Variable selection by random forests

We next trained a random forest on the recruited genomic features and further selected a small subset of variables based on the generated variable importance. In detail, each tree in the random forest is a bootstrap sample from the original data, and some observations are not in the bootstrap sample, called the out-of-bag (OOB) cases. For each tree, the prediction performance (measure by residual sum of squares) on the OOB proportion of data is recorded. The same procedure is done after permuting the values of each variable. Decrease of the prediction performance after permutation averaged over all the trees is taken as a measurement of variable importance. In this stage, we trained 25000 trees for the random forest. The variables were selected if their importance values are 2 * SD above the mean of all variable importance values.

### Quantile regression forests

We first denote the *τ*-th quantile of Y given *X* = *x* by *q*_τ_(*X*|*Y* = *x*). For *X* = *x*, the conditional distribution function *F*(*y*|*X* = *x*) is the probability that *Y* is smaller than or equal to *y* ∈ *R*, i.e.,

F(y|X=x)=P(Y≤y|X=x).

For a continuous distribution, the *τ*-th quantile *q*_*τ*_(*X*|*Y* = *x*) is defined as “*y*” such that *F*(*y*|*X* = *x*) = *τ*. While this definition cannot be extended to all cases, especially to discrete distributions, although the drug response (activity area) in this paper is continuous. In general, it is accepted that *q*_*τ*_(*Y*|*X* = *x*) = *inf*{*y*:*F*(*y*|*X* = *x*) ≥ *τ*}.

Quantile regression forests (QRFs) [17] estimate the conditional quantiles of response (*Y*) given the features (*X*) by building random forests. To build a QRF, a set of *T* un-pruned regression trees are generated based on bootstrap sampling from the original data. In this paper, we used *T* = 15000. For each node of the regression trees, a random set of *m* features selected from the whole set of *M* features is used for fitting a regression tree based on the bootstrap samples. In this paper, *m* was taken as *M*/3 as suggested by Hastie et al. [18]. In the tree generating process, a node with less than 10 training samples is not portioned any more [17]. Then the conditional distribution is estimated by the weighted distribution of the observed response variables. More specifically, we consider the conditional distribution of *Y* given *X* = *x* based on the tree *Ψ*. Suppose the leaf which contains *x* is denoted by *L*_*n*_(*x*,*Ψ*), then the weight *ω*_*i*_(*x*,*Ψ*) is given by

$$\omega_{i}(x,\Psi )=\frac{I(X_{i}\in L_{n}(x,\Psi ))}{\#\{j:X_{j}\in L_{n}(x,\Psi )\}}.$$

Let the *T* trees of the random forests be *Ψ*_1_.…,*Ψ*_*T*_, and *ω*_*i*_(*x*) be the average of *ω*_*i*_(*x*,*Ψ*) over all the trees. Then

$$\omega_{i}(x)=\frac{1}{T}\sum_{t=1}^{T}\omega_{i}(x,\Psi_{t}).$$

The estimated $\hat{F}(y|X=x)$ is then given by

$$\hat{F}(y|X=x)=\sum_{i=1}^{n}\omega_{i}(x)I(Y_{i}\leq y).$$ (1)

Then *τ*-th quantile *q*_*τ*_(*x*) is predicted by

$${\hat{q}}_{\tau }(Y|X=x)=inf\{y:\hat{F}(y|X=x)\geq \tau \}.$$

On the other hand, based on the generated trees in QRF, if we make a small change to the right side of formula (1), i.e., $\sum_{i=1}^{n}\omega_{i}(x)Y_{i}$, then the mean (expectation) of *Y* given *X* = *x* is predicted. So besides the conditional quantiles, QRFs can easily predict the conditional mean of response *Y*.

In this study, we implemented QRFs by R package “quantregForest” (version 0.2–3) and assessed the variable importance by permutation used in the original random forest algorithm.

### Prediction interval construction

The prediction intervals are constructed from the conditional quantiles of drug response predicted by QRFs. In detail, the (1 − α) × 100% prediction interval for drug response *Y* given genomic features *X* (a p-dimensional vector) is built by *I*(*x*) = [*q*_*α*/2_(*Y*|*X* = *x*), *q*_1−*α*/2_(*Y*|*X* = *x*)]. For example, the 95% prediction interval for drug response is estimated by

$$I(x)=[q_{0.025}(Y|X=x),\;q_{0.975}(Y|X=x)].$$

It means that for a given *x*, drug response locates in the interval *I*(*x*) with high probability. The length of the prediction interval fluctuates with *X*.

### Performance evaluation of prediction intervals

For each observation, we can obtain OOB prediction using the trees not containing this observation. OOB prediction is virtually equivalent to the prediction by cross validation when the number of trees is large [19]. In this study, we generated 15000 trees in QRFs and evaluated the prediction performance by QRFs using OOB prediction, avoiding the intensive computation of 10-fold cross validation [4]. For the point predictions of drug responses by QRFs, the Pearson correlation coefficients between the observed and predicted (OOB) values were used to quantify the accuracy. But the true conditional quantiles of drug responses are unobservable. So as suggested by Wang *et al*. [20], the prediction error of the *τ*-th conditional quantile was assessed based on the average of the value

$$\rho_{\tau }\left(Y-{\hat{q}}_{\tau }(Y|X=x)\right)$$

over all observations, where *ρ*_*τ*_(*r*) = τr − rI(r < 0) is called the quantile loss function, for 0 < *τ* < 1[10,20].

### Homogeneity test of variances for drug responses

Given the prediction intervals, we next consider prioritizing patients with very close point predictions of drug response to the same drug. As aforementioned, a shorter prediction interval indicates the higher stability of prediction at the same confidence level. Thus, the length of prediction interval reflects the prediction reliability. In this circumstance, patients with longer prediction intervals need further consideration and expect other medical plans. The strategy is intuitive but not statistically rigorous, so we used the homogeneity test of variances as a complement. Note that for each drug, we have only one response value for a specific patient (cell line), thus do not have enough replicates to carry out the homogeneity test directly. However, taking advantage of our random forest model, we have estimated the quantiles of drug response *q*_*τ*_(*Y*|*X* = *x*) for each cell line by the quantile regression forest. In detail, for any *τ* between *0* and *1*, we have obtained the estimate of *q*_*τ*_(*Y*|*X* = *x*). The quantile function of drug response equals to the inverse of cumulative distribution function since drug response is continuously distributed. This inspires us to use the inverse transform sampling to get the samples of drug responses [21]. In detail, for patient with geometric features *X* = *x*, we firstly generate different *τ*′*s* denoted as {*τ*_1_,..,*τ*_*k*_} from the standard uniform distribution *U*[0,1]. Then the corresponding $\left\{q_{\tau_{1}}(Y|X=x),\ldots \;q_{\tau_{k}}(Y|X=x)\right\}$ was taken as the random samples of drug responses for the considered patient. However, *q*_*τ*_(*Y*|*X* = *x*) is unknown, we thus treat $\left\{{\hat{q}}_{\tau_{1}}(Y|X=x),\ldots \;{\hat{q}}_{\tau_{k}}(Y|X=x)\right\}$ as the unobserved $\left\{q_{\tau_{1}}(Y|X=x),\ldots ,\;q_{\tau_{k}}(Y|X=x)\right\}$ as the random samples and carry out the rest analyses.

For simplicity, we denote ${\hat{q}}_{\tau }(Y|X=x)\;as\;Y^{*}$ in the remaining content. Assume that patients 1 and 2 have almost the same point predictions for a certain drug. By the inverse transform sampling, after randomly sampling *τ*_*ij*_′*s* from *U*[0,1] (*i* = 1,2,*j* = 1,…,*k*), we get $\left\{Y_{11}^{*},Y_{11}^{*},\ldots \;\ldots ,Y_{1k}^{*}\right\}$ and $\left\{Y_{21}^{*},Y_{21}^{*},\ldots \;\ldots ,Y_{2k}^{*}\right\}$ for patients 1 and 2, respectively. Then we consider the following hypothesis problem

$$H_{0}:\;\sigma_{1}^{2}=\sigma_{2}^{2}\;v.s.\;H_{1}:\;\sigma_{1}^{2}\neq \sigma_{2}^{2}.$$

Here $\sigma_{1}^{2}$ and $\sigma_{2}^{2}$ are the variances of drug responses for patients 1 and 2. We then use the Levene test [22], which is robust to the non-normal distributed samples, to test the difference of variances. Note that Levene test is not only restricted to two-group comparison, so we can also discuss the multi-patient test problem in the future. Similarly, the homogeneity test can also be used to compare different drugs for the same patient. In this paper, the significance level was set to *0*.*05*. We tried different values for *k*, and did not observe significant changes at different values of *k*. So we finally took *k* as 500.

## Results

### Quantile regression forests improve the accuracy of drug response prediction

**Fig 3:**
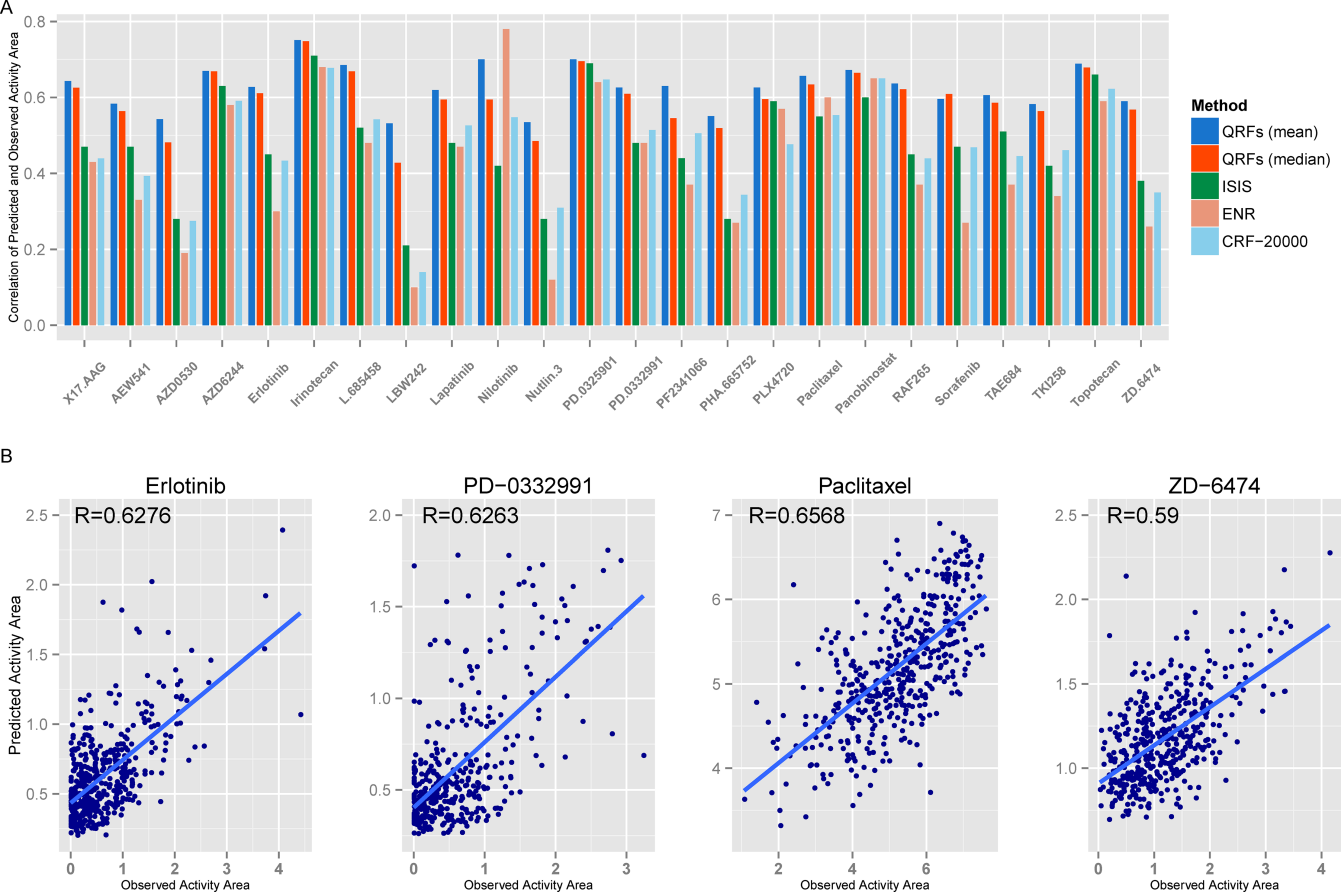
Prediction performance of quantile regression forests for CCLE data set. (A) Bar chart of Pearson correlation coefficients of drug responses and predicted values by QRFs, ENR, ISIS, and CRF-20000. QRFs (mean): (conditional) mean prediction of drug response given genomic features using QRFs; QRFs (median): median prediction of drug response using QRFs. (B) Scatter plots of observed and predicted drug responses (activity area) for four drugs in CCLE using QRFs.

We applied the three-step quantile regression forest (QRF) method to the CCLE dataset. Both the predicted mean and median of drug responses are taken as the point predictions for 24 drugs. The prediction accuracy quantified by the Pearson correlation coefficients of observed and predicted values, was reported in Table 1. Comparisons of QRFs with other methods including elastic net regression (ENR) [4], iterative sure independence screening (ISIS) [7] and weight-based integrated random forest with 20000 trees for CCLE data set (CRF-20000) [8] are shown in Fig 3A (also reported in S1 Table). We can observe that both the mean and median predictions by QRFs are much better than other approaches for most drugs. For QRFs, the accuracy of median prediction is slightly lower than mean prediction. Scatter plots of observed and predicted drug responses by QRFs (for mean) of 4 example drugs were drawn in Fig 3B. We conclude that the good correlations are fairly reasonable and not overestimated by a few outliers (Fig 3B).

**Table 1:** The Pearson correlation coefficients of observed and predicted drug responses (activity area) by QRFs.

Drug	mean	median	Drug	mean	median
**17−AAG**	0.6430	0.6255	**PD−0332991**	0.6263	0.6096
**AEW541**	0.5833	0.5637	**PF2341066**	0.6300	0.5455
**AZD0530**	0.5427	0.4818	**PHA−665752**	0.5509	0.5191
**AZD6244**	0.6697	0.6687	**PLX4720**	0.6261	0.5956
**Erlotinib**	0.6276	0.611	**Paclitaxel**	0.6568	0.6343
**Irinotecan**	0.7512	0.7478	**Panobinostat**	0.6721	0.6648
**L−685458**	0.6852	0.6688	**RAF265**	0.6366	0.6215
**LBW242**	0.5319	0.4279	**Sorafenib**	0.5960	0.6089
**Lapatinib**	0.6195	0.5947	**TAE684**	0.6059	0.5862
**Nilotinib**	0.7001	0.5943	**TKI258**	0.5826	0.564
**Nutlin−3**	0.5350	0.485	**Topotecan**	0.6889	0.6788
**PD−0325901**	0.7001	0.6951	**ZD−6474**	0.5900	0.5679

“mean” and “median” respectively represent the mean and median predictions of drug response by QRFs.

### Quantile regression forests construct prediction intervals

We next predicted the quantiles of drug response for the 24 drugs at different quantile levels, including *τ* = 0.025,0.1,0.25,0.5,0.75,0.9,0.975). S2 Table reported the prediction errors for different quantiles of drug response. The column labeled by “MPE” in S2 Table denotes the mean of prediction errors based on the quantile check loss function with the standard deviation reported in column “SD”. The prediction errors in S2 Table are very small, indicating good prediction performances for different quantiles. The detailed predicted quantiles of drug responses for 24 chemical compounds were listed in S3 Table. Based on the predicted quantiles, prediction intervals of drug response can be constructed (see details in Methods). Table 2 shows the average length and coverage probability of 95% and 80% prediction intervals. We find that most of the coverage probabilities are very close to 95% or 80%. This indicates the constructed prediction intervals are very reliable.

**Table 2:** Information of the 95% and 80% prediction intervals of drug responses for 24 drugs.

	95% PI		80% PI	
Drug	AveL	CP(%)	AveL	CP(%)
**17-AAG**	3.6591	96.2472	2.4550	0.8256
**AEW541**	1.9829	95.8057	1.8736	0.8102
**AZD0530**	2.4721	94.2731	1.7946	0.8260
**AZD6244**	3.3958	94.9227	1.9058	0.8124
**Erlotinib**	1.7705	93.1567	1.7617	0.8102
**Irinotecan**	3.3840	96.4413	1.8078	0.8221
**L-685458**	1.2236	81.6327	1.6639	0.7029
**LBW242**	1.9626	94.7020	1.5935	0.7837
**Lapatinib**	1.8360	92.0705	1.5459	0.7996
**Nilotinib**	1.8769	93.3333	1.5041	0.7920
**Nutlin.3**	1.4540	93.6123	1.4501	0.8106
**PD-0325901**	4.2187	94.9339	1.5704	0.8128
**PD-0332991**	1.7023	91.5167	1.5412	0.8021
**PF2341066**	1.7343	95.1542	1.5056	0.7753
**PHA-665752**	1.6059	89.8455	1.4727	0.8079
**PLX4720**	1.4942	90.3803	1.4398	0.7964
**Paclitaxel**	4.1470	95.3642	1.5183	0.8146
**Panobinostat**	2.3412	94.6667	1.5209	0.8022
**RAF265**	2.5618	95.8838	1.5291	0.8208
**Sorafenib**	1.4507	95.1435	1.5004	0.7991
**TAE684**	2.7567	95.5947	1.5141	0.8040
**TKI258**	1.8532	94.2731	1.5003	0.8194
**Topotecan**	3.8207	96.0352	1.5456	0.8150
**ZD-6474**	2.3895	95.5257	1.5464	0.8210

"95% PI" and "80% PI" denote the 95% and 80% prediction intervals; "AveL" stands for the average length of prediction intervals; "CP" denotes the coverage probability.

### Prediction reliability assessed by prediction intervals provides more information for precision medicine

In contrast to point prediction with a single value and no information about the possible fluctuations of drug response, prediction interval gives a range containing the drug response with a high probability and assess the reliability by its length. At a certain given confidence level, shorter prediction interval indicates less fluctuations of drug response and hence means more reliable drug. Thus a drug with shorter prediction interval may possibly beat another one with longer prediction interval, especially when the point predictions are very close to each other. Also, for the same drug, it is more appropriate to distinguish the candidate patients with close point predictions but quite different prediction intervals. Since the patients with longer prediction intervals are inclined to have more instable curative effect, it should be better for them to try other drugs. We want to point out that evaluation of drug efficacy based on the length of prediction intervals is intuitive but not statistically strict. In this paper we also proposed a homogeneity test of variances for drug responses to provide more statistical evidences.

Besides the reliability inflected by the length of prediction interval, another layer of reliability lies in the upper and lower ends (prediction limits) of the interval. For example, the upper end of the 95% prediction interval is the 97.5%-th quantile prediction of drug response, which means that the drug response may exceed the upper end with a probability around 2.5%; similarly, the lower end is the 2.5%-th quantile prediction, which means the drug response can outperform the lower end with a probability around 97.5%. Therefore, this layer of reliability can offer guidelines to different orientations of medical treatments. To be specific, the drug with the highest upper prediction limit is more likely to give an aggressive treatment; while the drug with the highest lower prediction limit may be a good choice as a conservative plan.

So, in summary, prediction reliability assessed by prediction intervals can provide more information for precision medicine. In order to explain this more clearly, we then give two examples from CCLE dataset as follows.

**Fig 4:**
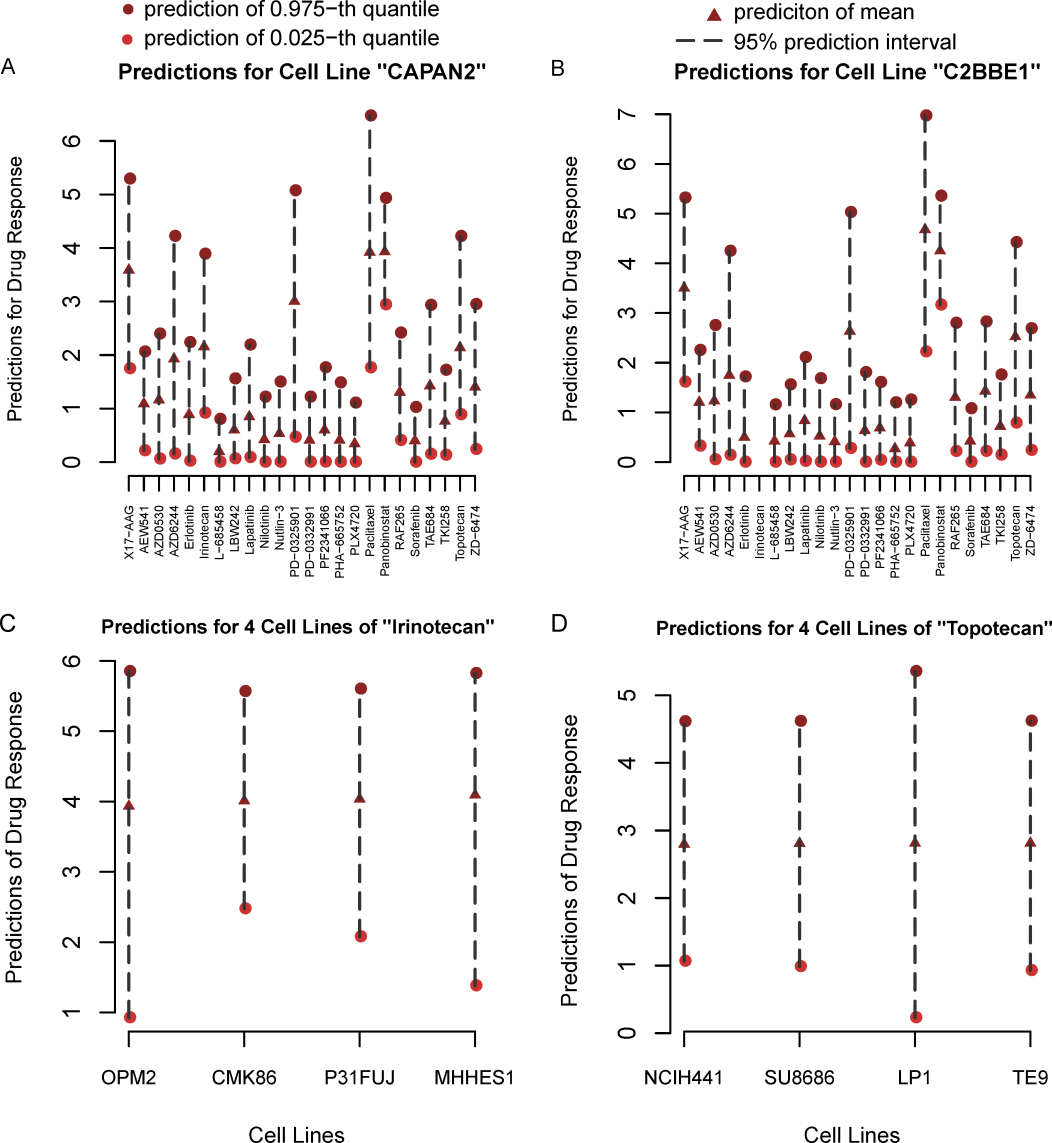
The 95% prediction intervals and mean predictions by quantile regression forests. Red triangular indicates the point (or mean) prediction of drug response, two red dots indicates the upper and lower boundaries of 95% prediction interval. (A) and (B) show the comparisons of 24 drugs for cell lines “CAPAN2” and “C2BBE1”, respectively. (C) and (D) are the comparisons of four different cell lines to drugs “Irinotecan” and “Topotecan”, respectively.

**Example A.** In this example, we explore the potential treatment choices, Paclitaxel and Panobinostat, for the patient “CAPAN2” and “C2BBE1” due to their high predicted drug response. As is shown in Fig 4A, both drugs show very small difference in terms of the point predictions. But actually, Panobinostat shows a shorter prediction interval compared to Paclitaxel and the homogeneity test of variances for these two drugs brings the p-value less than 2.2 × 10^−16^. So Panobinostat is preferred due to its more stable curative effect. Similarly, in Fig 4B for patient “C2BBE1”, Paclitaxel is the better choice due to its highest mean prediction of drug response if the fluctuations of drug response are neglected. But Panobinostat, which has the second highest point prediction, gives much shorter 95% prediction intervals than Paclitaxel. Moreover, the p-value of homogeneity test of variances for Paclitaxel and Panobinostat is much less than 0.001. Thus, Panobinostat should be a better decision if the stability of treatment effect is taken to the consideration. Furthermore, Paclitaxel possesses a higher predicted upper limit and lower limit compared to Panobinostat for both patients (Fig 4A and 4B). Thus, the better drugs may be different for different purposes. Generally speaking, Panobinostat is applicable for a conservative treatment, while Paclitaxel is more risky and aggressive.

By this example, we concluded that prediction intervals provide more suggestions for therapeutic strategies. Prediction intervals can help to make more sensible decisions based on the expectation of the curation.

**Example B.** In this example, we discuss the response of different patients treated with the same drug. As shown in Fig 4C, due to the closer mean predictions of drug responses, four patients can be classified together if prediction intervals are not considered. However, if prediction intervals are taken into account, patients “OPM2” and “MHHES1” have relatively longer prediction intervals than “CMK86” and “P31FUJ”. To further validate this result, we took the homogeneity tests of variances for the drug responses of these patients. The difference between “CMK86” and “P31FUJ” is not significant (p-value = *0*.*8717*). But if “OPM2” or “MHHES1” is incorporated, the p-value of homogeneity test becomes less than *0*.*001*. Both prediction intervals and homogeneity tests indicate the treatment effects for “OPM2” and “MHHES1” are highly variable. So it is better to consider other drugs for patients “OPM2” and “MHHES1”, and the four patients are then distinguished. A similar example was shown in Fig 4D where the 95% confidence interval of patient “LP1” is the longest. The homogeneity test of variances for the other three patients is not significant with *p*-value *0*.*7686*, while the test shows high significance with the *p*-value 2.569 × 10^−13^ when all the four patients were incorporated. This example clarifies that the patients who are grouped together by point predictions may be possibly distinguished when considering prediction reliability of drug response. So we conclude that prediction intervals are useful to determine a better personalized treatment.

### Functional annotations of genes used by quantile regression forests

**Fig 5:**
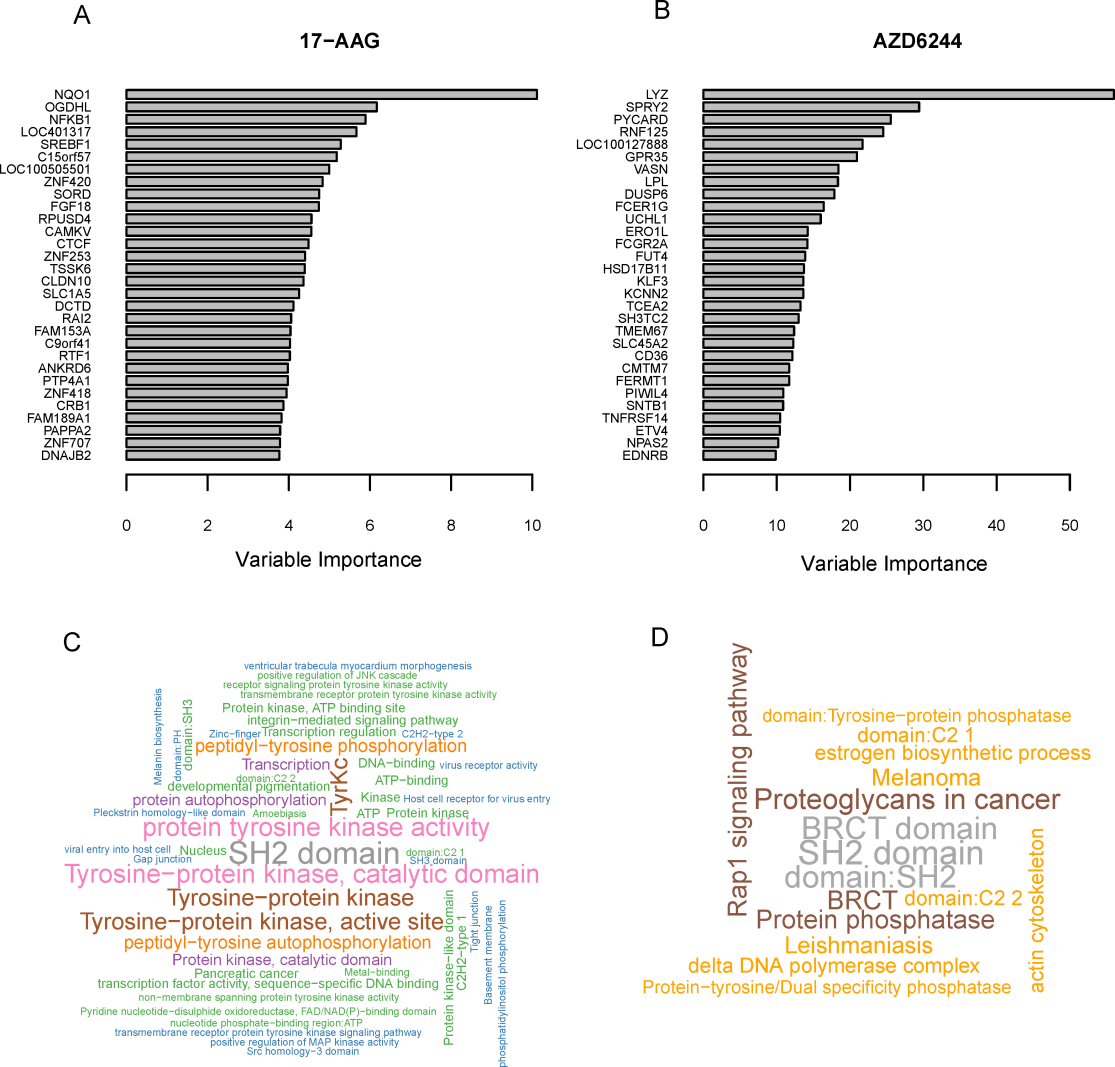
Variable importance and word clouds of functional annotations for the genes used by QRFs. Panels (A) and (B) are the bar charts of variable importance for drugs 17-AAG and AZD6244. Word clouds of functional annotations of the genes for 24 drugs are in panel (C) (all genes) and panel (D) (ensemble of top 30 genes of each drug), where font size of each annotation indicates its enrichment score.

The genes used by quantile regression forests (QRFs) for 24 drugs were listed in S4 Table, ranked by their importance for predicting the conditional mean of drug response. We drew the bar plots of the importance of the top 30 gene signatures in Fig 5A and 5B for 17-AAG and AZD6244, respectively. In S4 Table, many genes used by QRFs are pointed out to be related with cancers in literatures. For example, the inhibition activity of 17-AAG can be increased by the expression of *NQO1*[23]. Also, the mutation of *BRAF* is predicted as a drug efficacy marker for some MEK inhibitors, including AZD6244, PD-0325901 and PLX4720 [24]. These genes were also detected by elastic net regression and iterative sure independence screening [4,7].

Besides the aforementioned gene signatures, many other drug response related genes which were not selected by Barretina et al. 2012 [4] or Fang et al. 2015 [7] were also detected by our study. For example, genes *OGDHL*, *NFKB1*, *LOC401317*, and *FGF18* are among the top 10 genes of drug 17-AAG. It has been pointed out that the re- expression of *OGDHL* can induce apoptosis in cervical cancer cells [25]. Also, people have detected a significant alteration in expression of *NFKB1* in adenocystic carcinomas, which suggests that *NFKB1* might be served as a target for innovative diagnostic and treatment programs [26]. In addition, *LOC401317* could induce apoptosis in the nasopharyngeal carcinoma cell line HNE2 [27], and *FGF18* (Fibroblast growth factor 18) is a prognostic and therapeutic biomarker for ovarian cancer [28].

The drug response related genes of all the 24 drugs were assembled and annotated by David tools (https://david.ncifcrf.gov/summary.jsp). Word cloud plots of functional annotations (FDR<0.1) were drawn in Fig 5C and 5D. Fig 5C demonstrates the functional annotations of all the genes, and Fig 5D refers to the top 30 genes of 24 drugs. We can see that many annotations have close relationship with cancers. For example, “SH2 (Src Homology 2) domain” is the most significant term in both Fig 5C and 5D. “SH2 domain”, a structurally conserved protein domain, is important to the treatment of breast cancer [29], the EGFR inhibitor Erlotinib, and the SRC/multi-kinase inhibitor Dasatinib of lung cancer [30]. Moreover, “tyrosine” and “protein kinase” appear frequently in Fig 5C, such as in the terms “tyrosine-protein kinase, catalytic domain”, “protein tyrosine kinase activity”, “serine-threonine/tyrosine-protein kinase catalytic domain” and “receptor signaling protein tyrosine kinase activity” etc. It has been discovered that receptor tyrosine kinases (RTK), play key roles in growth, metabolism, adhesion, motility, death and oncogenesis [31]. Also, protein kinases are critical in many cellular processes, including division, proliferation, apoptosis, and differentiation [32]. In addition, Src-family of protein-tyrosine kinases are also related with oncogenesis, proliferation, and survival[33]. In Fig 5D, besides the “SH2 domain” mentioned above, “BRCT domain” (after the C_terminal domain of a breast cancer susceptibility protein) and “Proteoglycans in cancer” are also top terms. These results show that the drug response related genes involve in various biological activities of carcinomas.

## Discussion and conclusion

In this paper, we proposed a three-step quantile regression forest (QRF) method to give point and interval predictions of drug response. The method was applied to the CCLE dataset, modeling on the genomic features including baseline gene expressions, mutation status and copy number variations. The contribution of our method is two-fold. First, we gave a point estimation of drug sensitivity prediction based on random forest model. Second, we gave the prediction confident interval based on the quantile regression forest, which is helpful when point estimations of two drugs to a patient are very similar. By a series of examples, we state that the prediction intervals can help to choose different therapeutic regimens from different orientations, such as the preference of the stability of medical effect, or a radical plan or a conservative curation. Such kind of information could further help researchers to determine the best clinical strategy for a specific patient in some circumstance.

In order to evaluate the difference between two prediction intervals, we also proposed a heterogeneity test of variance among patients and differentiating them through the lengths of prediction intervals supplemented by the homogeneity test. By constructing prediction intervals complemented with homogeneity test of variances, our paper brings a new perspective to acknowledge and adopt the prediction reliability which is important but usually ignored in precision medicine. Hereby we also want to point out that the proposed quantile regression forest based method is applicable for prediction problems with high dimensional covariates in extensive scientific and social fields, not limited to drug response prediction.

Before building the quantile regression forest, we used Pearson correlation coefficient to screen all possible features. Actually, besides Pearson correlation coefficient, there are many other measures that could be used to rank the marginal importance of features to drug response, such as generalized correlation [34], rank correlation [35], distance correlation [36], etc. For review of this field, please refer to Liu *et al*. [37]. We chose the Pearson correlation for feature screening mainly due to its straightforward implementation and popularity in drug response prediction [4, 5].

There are still several shortcomings that should be further explored in the future. First, the QRFs improved the prediction accuracy by assembling a bunch of regression trees but lost the interpretability as the price. In other words, we cannot give a clear model with explicit regression coefficients by this study. A better predictor of drug response with good interpretability will be our goal in the future. Second, in this paper, we compared the drug response reliability mainly by statistical inferences, including the prediction intervals and the homogeneity test of variances but without real experiments for further validations. The experimental validations need to treat a series of cell lines (or patients) for multiple times simultaneously by a same set of drugs. Due to our present research conditions, we cannot carry out these experiments. We hope our results could motivate other experimental researchers to conduct such kind of experiments in the future.

## Supporting information

S1 Table Pearson correlation coefficients of real and predicted drug responses by QRFs, ISIS, ENR and CRF-20000. (XLSX)

S2 Table Prediction errors based on the quantile loss function of different *τ*-th quantiles of drug responses by quantile regression forests. (XLSX)

S3 Table Observations and predictions of drug responses of 24 drugs for all the cell lines. (XLSX)

S4 Table Genes used by quantile regression forests and the generated variable importance. (XLSX)

S1 Dataset Drug response data. (XLSX)

S2 Dataset Mutation data. (TXT)
